# From Intention to Enactment: Action Planning and Habit Automaticity Distinguish Successful from Unsuccessful Intenders to Engage in Regular Leisure-Time Moderate-to-Vigorous Physical Activity

**DOI:** 10.3390/bs16060989

**Published:** 2026-06-15

**Authors:** Yaogang Han, Yubing Wang, Pan Li, Binn Zhang

**Affiliations:** 1 School of Physical Education, Shanghai University of Sport, Shanghai 200438, China; hanyaogang@sus.edu.cn; 2 Department of Human Movement Studies & Special Education, Old Dominion University, Norfolk, VA 23529, USA; y8wang@odu.edu; 3 School of Athletic Performance, Shanghai University of Sport, Shanghai 200438, China; lipan@sus.edu.cn; 4 School of Psychology, Shanghai University of Sport, Shanghai 200438, China

**Keywords:** physical activity, intention-behavior gap, action planning, coping planning, habit automaticity, university students

## Abstract

University students often intend to exercise regularly but fail to translate intention into action. The present study tested which post-intentional processes distinguish successful from unsuccessful intenders in self-reported regular leisure-time moderate-to-vigorous physical activity (MVPA) enactment. Chinese undergraduates from 10 universities completed a three-wave survey administered at roughly two-week intervals. Habit automaticity was assessed at Wave 1, intention together with action planning and coping planning at Wave 2, and self-reported regular-exercise status at Wave 3. Because the dependent variable was assessed using a single stage-based self-report item, the findings should be interpreted as explaining self-reported regular-exercise status rather than objectively measured MVPA volume, frequency, or intensity. Primary analyses focused on students classified as intenders under the prespecified threshold (*n* = 1119 of N = 1670) and used hierarchical logistic regression to predict Wave 3 active versus inactive status. Under the primary threshold, 43.23% of participants were successful intenders and 23.77% were unsuccessful intenders, yielding an intention-behavior gap of 35.48% among intenders. Confirmatory factor analyses supported treating action planning and coping planning as distinct constructs. Among intenders, stronger action planning, stronger habit automaticity, and stronger intention strength independently predicted greater odds of meeting the regular-exercise criterion at follow-up. Coping planning did not show unique predictive value once action planning, habit automaticity, and intention strength were considered simultaneously, and no planning × habit interaction was supported. The pattern was robust across three alternative intention thresholds. These findings suggest that, among already motivated university students, successful exercise enactment depends less on coping planning alone than on a combination of commitment, concrete scheduling, and emerging behavioral automaticity. Interventions for student physical activity may therefore benefit from emphasizing detailed action planning and repeated performance in stable contexts.

## 1. Introduction

Regular leisure-time moderate-to-vigorous physical activity (MVPA) is a core health behavior during emerging adulthood. Current physical activity guidelines recommend that adults accumulate 150–300 min of moderate-intensity aerobic activity, 75–150 min of vigorous-intensity activity, or an equivalent combination each week for substantial health benefits (Bull et al., 2020). Insufficient physical activity is a global public health concern across adulthood, but young adulthood is especially important because activity routines often become less externally structured and more dependent on personal planning, social contexts, and daily life demands (Strain et al., 2024). University students share several challenges with same-aged non-student young adults, including increasing autonomy, changing social networks, competing academic or work-related demands, and screen-based leisure. However, the university context is also distinct: students often have access to campus sport and fitness resources, but their exercise routines may also be disrupted by academic schedules, examination periods, shared living environments, peer norms, and sedentary study demands. The university years are therefore an important period for establishing repeated leisure-time exercise routines because students begin to manage their own schedules, living contexts, and health behaviors more independently.

Yet university students remain a population for whom sustained exercise can be difficult to establish and maintain. International evidence also suggests that this is not a narrow local issue: a systematic review of high school and university students across multiple countries identified lack of time, lack of motivation, and lack of accessible places as recurring barriers to physical activity (Ferreira Silva et al., 2022). A recent systematic review using the Theoretical Domains Framework and COM-B concluded that university students’ physical activity is shaped most consistently by environmental context and resources, social influences, and goals, suggesting that student activity is constrained by competing demands and self-regulatory challenges rather than by motivation alone (Brown et al., 2024).

This issue is particularly relevant among Chinese university students. Prior evidence suggests that physical inactivity among Chinese university students may be higher than in the general Chinese adult population, with reports of approximately 51.5% physical inactivity and 8.5–9.5 h/day of sedentary behavior (Yu & Ye, 2023). In a recent six-region survey of 11,173 Chinese university students, the proportion reporting more than 60 min/day of MVPA was substantially higher among male students than female students, 8.2% versus 2.3%, respectively, highlighting a potential sex difference in high-volume MVPA participation (Deng et al., 2024). These patterns may reflect contextual features of Chinese university life, including academic pressure or burden, screen-based study and leisure, shared campus living arrangements, and campus environmental opportunities for exercise (Ma et al., 2024; Zheng et al., 2024). At the same time, intervention evidence indicates that physical activity can be improved in university students, but effect heterogeneity remains substantial (Yuan et al., 2024). Together, these findings suggest that the key problem is not simply whether students value exercise, but why some students who intend to be active follow through, whereas others do not.

This question lies at the center of the physical activity intention-behavior gap literature. Behavioral intention is a central proximal predictor in social-cognitive models, yet intention alone does not reliably translate into action. A recent systematic review and meta-analysis estimated an overall physical activity intention-behavior gap of 47.6%, meaning that a large proportion of people who intend to be active nevertheless fail to enact those intentions (Feil et al., 2023). Similarly, Rhodes et al. concluded that the intention-physical activity relationship depends on additional moderators, particularly reflective and automatic processes (Rhodes et al., 2022). Understanding exercise behavior therefore requires moving beyond intention as a stand-alone predictor toward mechanisms that explain intention enactment.

The Health Action Process Approach (HAPA) provides one influential account of this problem. HAPA distinguishes between a motivational phase, in which intentions are formed, and a volitional phase, in which intentions are translated into behavior (Schwarzer, 2008). Within this volitional phase, planning has been conceptualized as a key self-regulatory mechanism bridging intention and action. A meta-analysis of the HAPA literature supported the distinction between motivational and volitional determinants and reinforced the importance of post-intentional processes in health behavior enactment (Zhang et al., 2019). More specifically, action planning involves specifying when, where, how, and how often a behavior will be performed, whereas coping planning involves anticipating obstacles and identifying strategies to overcome them. Longitudinal work in physical activity has shown that planning can help explain how intentions are translated into subsequent behavior (Scholz et al., 2008).

Although HAPA is highly useful for explaining volitional processes, recent theory in physical activity has emphasized that self-regulation alone is unlikely to fully explain stable exercise behavior. The Multi-Process Action Control (M-PAC) framework argues that physical activity reflects layered reflective, regulatory, and reflexive processes, and that regulatory tactics help maintain concordance between reflective motivation and behavior until more reflexive processes, such as habit, begin to co-determine action control (Rhodes, 2021). This emphasis on reflexive processes is consistent with broader habit theory, which conceptualizes habit as cue-triggered automaticity that develops through repeated behavioral performance in stable contexts (Gardner, 2015). More recent theoretical work has likewise argued that the next generation of physical activity behavior-change research needs more precise attention to behavior change itself and to the automatic processes that support maintenance and enactment (Simpson et al., 2025). A model that jointly considers planning and habit automaticity is therefore well suited to explaining why some student intenders succeed whereas others do not.

At the same time, the relative roles of action planning and coping planning remain unsettled. A meta-analysis by Carraro and Gaudreau supported positive roles for both forms of planning in physical activity (Carraro & Gaudreau, 2013), whereas a more recent meta-analytic structural equation modeling study suggested that coping planning may be more important than action planning for translating exercise intention into behavior (Wang et al., 2025). Yet this issue has not been tested consistently in prospective studies focused on university students, nor in designs that simultaneously model prior-wave habit automaticity. This gap is important because student exercise is often embedded in fluctuating schedules, competing academic demands, and inconsistent routines, all of which may alter the relative importance of concrete scheduling versus obstacle management.

The need for such work is particularly evident in Chinese college student research. Existing studies in this population have shown that planning processes matter, but they have tended to address narrower questions. For example, Hou et al. used a six-wave design to show that both action planning and coping planning were implicated in the exercise intention-action link under higher self-efficacy (Hou et al., 2022), whereas Zhu et al. examined action planning and habit in a moderated mediation model of Chinese college students’ exercise behavior (Zhu et al., 2022). Related prior work by members of the present author team also examined attitude, habit strength, and the intention–leisure-time MVPA relationship among college students, but it did not compare action planning with coping planning or classify students into successful versus unsuccessful intenders (Han et al., 2025). These studies are informative, but they do not resolve a more focused action-control question: among students who already intend to exercise regularly, which post-intentional processes uniquely distinguish successful from unsuccessful intenders when action planning, coping planning, and habit automaticity are considered together? Moreover, few studies have addressed this question using a behaviorally matched regular-exercise criterion assessed prospectively across waves.

The present study addressed this gap by testing a partial volitional action-control model of regular leisure-time moderate-to-vigorous physical activity (MVPA) in Chinese undergraduates. The three-wave design was used to approximate the temporal sequence implied by action-control theory: prior habit automaticity as an indicator of established routine strength, subsequent intention and planning as post-intentional volitional processes, and later regular-exercise status as the enactment outcome. This design also reduced the limitations of assessing all focal constructs at a single time point and allowed a more prospective test of which post-intentional processes distinguish successful from unsuccessful intenders. We examined whether Wave 2 action planning and coping planning and Wave 1 habit automaticity distinguished which students later met a matched regular-exercise criterion at Wave 3. We also quantified action-control profiles—successful intenders, unsuccessful intenders, non-intenders who remained inactive, and non-intenders who were nevertheless active—and evaluated whether the conclusions were robust across alternative intention thresholds. This classification directly matched the study question because the central issue was not simply whether intention predicted behavior, but which post-intentional processes distinguished intenders who later reported regular exercise from intenders who did not. Guided by HAPA and M-PAC, we expected that stronger planning and stronger habit automaticity would be associated with successful exercise enactment. Because the literature is mixed regarding the comparative value of action planning versus coping planning, and because habit automaticity may potentially modify planning effects, these were treated as empirical questions.

## 2. Materials and Methods

### 2.1. Study Design, Participants, and Procedure

**Figure 1:**
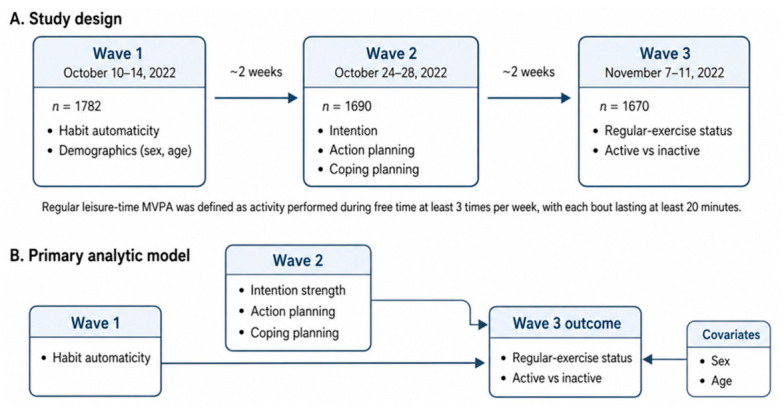
Three-wave research design and primary analytic model. Panel (**A**) summarizes the timing, sample size, and variables measured at each survey wave. Panel (**B**) summarizes the primary analytic model used to predict Wave 3 regular-exercise status among intenders. Primary inferential analyses focused on participants classified as intenders under the primary threshold of mean intention ≥4.0. MVPA = moderate-to-vigorous physical activity.

This study followed Chinese undergraduate students across three survey waves administered during the fall semester of 2022 at approximately two-week intervals. Wave 1 data were collected approximately during 10–14 October 2022; Wave 2 data were collected approximately during 24–28 October 2022; and Wave 3 data were collected approximately during 7–11 November 2022. The three-wave structure was designed to separate baseline reflexive action-control resources, post-intentional volitional processes, and later self-reported behavioral enactment. Accordingly, habit automaticity and demographic information were collected at Wave 1, intention together with action planning and coping planning were measured at Wave 2, and regular exercise status was assessed at Wave 3. In the survey, regular leisure-time MVPA was defined as engaging in moderate-to-vigorous physical activity during one’s free time at least three times per week, with each bout lasting at least 20 min. This definition served as the target behavior across the leisure-time MVPA items, including intention, planning, habit automaticity, and Wave 3 regular-exercise status. Importantly, MVPA was not analyzed as a separate continuous variable in the Results; instead, the behavioral outcome was whether participants reported currently meeting this regular leisure-time MVPA criterion. The three-wave study design and primary analytic model are summarized in Figure 1.

Participants were recruited from 10 universities through the researchers’ personal and institutional networks. Questionnaires were distributed during obligatory university classes, but participation in the study itself was voluntary and was not part of the course assessment. Students were informed before data collection that they could decline participation, skip any item, or withdraw from the study at any time without penalty or negative consequences. The number of students who declined participation before completing the Wave 1 questionnaire was not systematically recorded; therefore, refusal at initial recruitment could not be quantified. All three waves were administered using paper-and-pencil questionnaires. Ethical approval for the study was granted by the Scientific Research Ethics Review Committee of Shanghai University of Sport. Participation was voluntary, and informed consent was obtained before data collection. Because the study followed participants across three waves, responses were matched using each participant’s unique university student ID number. The student ID was used only for longitudinal matching across waves. After matching was completed, ID numbers were removed from the analytic dataset, and all analyses were conducted using de-identified data. No student names were retained in the analytic dataset. Thus, the data were treated as confidential during longitudinal matching and de-identified before analysis, rather than fully anonymous at the time of collection. At Wave 1, 1782 students completed the survey; 1690 of these participants also completed Wave 2, and 1670 completed Wave 3. These figures correspond to retention rates of 94.8% from Wave 1 to Wave 2, 98.8% from Wave 2 to Wave 3, and 93.7% across all three waves. The final analytic sample therefore included 1670 students with complete data on all focal variables. The final analytic sample included 752 female students (45.0%) and 918 male students (55.0%), with a mean age of 20.05 years (SD = 1.02).

### 2.2. Measures

Reliability and construct validity evidence for the multi-item measures was evaluated in the present sample. The response format for each construct was retained from the source or established physical activity measures cited below, rather than harmonized across scales, to preserve item meaning and comparability with prior research using these instruments. Internal consistency was strong across the scales (Cronbach’s α = 0.90–0.93), composite reliability ranged from 0.93 to 0.95, and CFA model comparisons supported the distinctiveness of the focal constructs; detailed coefficients and model-fit indices are reported in Table 1 and Supplementary Table S1. To improve measurement transparency, Supplementary Table S1, Panel C now reports the Chinese item wording administered in the study, author-provided English translations, item-level descriptive statistics, and standardized factor loadings for the focal multi-item measures, with response-scale information provided in the table note.

#### 2.2.1. Habit Automaticity

Habit automaticity was assessed using the four-item automaticity subscale of the Self-Report Habit Index, also known as the Self-Report Behavioural Automaticity Index (SRBAI) (Gardner et al., 2012). The items captured the extent to which leisure-time MVPA was experienced as automatic, enacted without conscious thought, and initiated before conscious awareness. For example, one item asked whether leisure-time MVPA was something participants did automatically. Thus, the scale assessed the psychological automaticity of leisure-time MVPA rather than the frequency, duration, intensity, or total amount of MVPA itself. Responses were made on a 7-point scale, and the scale score was computed as the mean of the four items. Gardner et al. showed that the SRBAI provides a parsimonious measure of the automaticity component of habit and is sensitive to both habit-behavior associations and the moderation of the intention-behavior relationship (Gardner et al., 2012).

#### 2.2.2. Intention

Intention was assessed with three items referring to the next month of regular leisure-time MVPA. The items assessed participants’ intention, plan, and perceived likelihood of performing regular leisure-time MVPA over the next month. In this scale, the item using the wording “plan” was treated as an intention-strength item, reflecting a global motivational plan or commitment to perform regular leisure-time MVPA over the next month. It did not ask participants to specify implementation details such as when, where, what type of activity, or how often they would exercise. Responses were made on a 6-point scale, and the scale score was computed as the mean of the three items. This operationalization is consistent with prior physical activity work treating intention as both a decisional and a strength-related construct (Rhodes & Rebar, 2017) and with earlier planning research in physical activity (Scholz et al., 2008; Sniehotta et al., 2006).

#### 2.2.3. Action Planning

Action planning was assessed using four items capturing whether participants had made detailed plans regarding when, where, what type of activity, and how often they would perform leisure-time MVPA. Thus, action planning was operationally distinguished from intention by its focus on implementation specificity rather than motivational commitment. Whereas the intention items assessed whether students intended, planned, or expected to engage in regular leisure-time MVPA, the action-planning items assessed whether students had translated that motivation into concrete behavioral specifications. Responses were made on a 5-point scale, and the action-planning score was computed as the mean of the four items. This operationalization follows the classic HAPA distinction between intention and concrete implementation details (Schwarzer, 2008; Scholz et al., 2008; Sniehotta et al., 2006).

#### 2.2.4. Coping Planning

Coping planning was assessed using three items measuring whether participants had made detailed plans for handling disruptions to their exercise plans, anticipated obstacles, and situations in which carrying out their plans would be difficult. Responses were made on the same 5-point scale, and the coping-planning score was computed as the mean of the three items. Consistent with HAPA, coping planning was conceptualized as anticipatory self-regulation aimed at overcoming barriers to intended action (Schwarzer, 2008; Scholz et al., 2008; Sniehotta et al., 2006).

#### 2.2.5. Regular-Exercise Status

Regular-exercise status was assessed using a single self-reported stage-based item derived from the transtheoretical model tradition of exercise behavior change (Prochaska & DiClemente, 1983). Participants were asked to select the statement that best described their current regular leisure-time MVPA status, using the study definition of regular leisure-time MVPA as leisure-time moderate-to-vigorous activity performed at least three times per week for at least 20 min per bout. The response options distinguished students who were not currently engaging in regular leisure-time MVPA and had no intention to begin, those who were not currently engaging but intended to begin, those currently engaging for less than 6 months, and those currently engaging for more than 6 months. Thus, the item did not use a fixed past-week or past-month recall period; rather, its temporal reference was current regular-exercise status, with current exercisers further distinguished by whether they had maintained the behavior for less than or more than 6 months.

The response options reflected the standard distinction among individuals who were currently engaging in regular exercise, those who had recently adopted regular exercise, and those who were not currently exercising regularly but differed in their readiness to begin (Marcus & Simkin, 1993). For the purposes of the present study, participants were classified as active if they endorsed either current regular exercise for more than 6 months or current regular exercise for less than 6 months and as inactive if they reported not currently exercising regularly, regardless of whether they planned to begin in the near future. This dichotomous coding was used because the study focused on the intention–behavior gap, which requires classifying participants according to whether they intended to perform the target behavior and whether they later reported meeting the corresponding behavioral criterion. This coding matched the study’s central question, whether students were currently meeting the same regular-exercise criterion targeted by the intention items, rather than finer distinctions among inactive stages.

Thus, the dependent variable should be understood as a self-reported regular-exercise status indicator. This behavioral classification was distinct from the SRBAI habit measure: regular-exercise status classified whether students reported meeting the regular-exercise criterion at Wave 3, whereas habit automaticity assessed the subjective automaticity of leisure-time MVPA at Wave 1. It did not provide objective information on MVPA frequency, duration, intensity, total volume, or accelerometer-derived activity. Stage-of-exercise measures of this type have shown meaningful associations with exercise behavior and related correlates (Dannecker et al., 2003).

### 2.3. Data Reduction and Group Classification

Scale scores for habit automaticity, intention, action planning, and coping planning were computed as arithmetic means of their constituent items. Under the primary threshold, a participant was classified as an intender if the mean intention score was ≥4.0 on the 1–6 scale. This threshold was chosen because 4 represented the first clearly positive response category on the scale and therefore approximated a positive decisional intention while retaining variation in strength of intention. Because any threshold-based definition of intention involves some judgment, three robustness checks were also specified: a more inclusive threshold (mean intention >3.5), a more conservative threshold (mean intention ≥5.0), and a single-item decisional threshold based on the first intention item (Intention item 1 ≥ 4.0).

For descriptive action-control analyses, participants were cross-classified by intention status and regular-exercise status into four groups: successful intenders, unsuccessful intenders, non-intenders who were active, and non-intenders who were inactive. Successful intenders were students who met the intention threshold and later reported regular exercise, whereas unsuccessful intenders were students who met the intention threshold but did not later report regular exercise. These two groups were central to the primary research question because they allowed us to examine which post-intentional processes distinguished successful from unsuccessful enactment among students who already intended to exercise regularly. Active and inactive non-intenders were retained descriptively to show the full intention–behavior classification in the total sample. This action-control classification approach is consistent with recent physical activity intention gap research (Feil et al., 2023).

### 2.4. Statistical Analysis

All analyses were conducted in R version 4.5.0 using haven, dplyr, psych, and lavaan (Rosseel, 2012). To evaluate potential attrition bias, participants included in the final analytic sample were compared with participants excluded because they did not complete all focal variables across the three waves. Because intention and planning were assessed at Wave 2 and were therefore unavailable for participants lost after Wave 1, the primary attrition analysis focused on baseline characteristics available for the full Wave 1 sample: sex, age, and Wave 1 habit automaticity. Sex differences were examined using a chi-square test, and age and habit automaticity were compared using independent-samples *t* tests. Effect sizes were summarized using Cramér’s V for sex and Cohen’s d for continuous variables. Internal consistency was assessed using Cronbach’s alpha, and composite reliability estimates were derived from confirmatory factor analysis (CFA) models. CFAs were estimated using the weighted least squares means and variance adjusted estimator (WLSMV), which is appropriate for ordered categorical indicators and is widely recommended for CFA with ordinal data (Flora & Curran, 2004; Li, 2016). Two planning models were compared: a one-factor model in which all seven planning items loaded on a single latent factor and a two-factor model in which action-planning and coping-planning items loaded on separate but correlated factors. In addition, a full three-factor model (habit, intention, combined planning) was compared with a full four-factor model (habit, intention, action planning, coping planning). Model fit was evaluated using the comparative fit index (CFI), Tucker–Lewis index (TLI), root mean square error of approximation (RMSEA), and standardized root mean square residual (SRMR), with emphasis on the overall fit pattern rather than rigid adherence to any single cutoff (Hu & Bentler, 1999).

Zero-order associations were examined using Pearson correlations. Because activity status was binary, correlations involving the outcome can be interpreted as point-biserial associations. Primary inferential analyses focused on participants classified as intenders under the primary threshold and used binary logistic regression to predict Wave 3 self-reported active versus inactive status. Non-intenders were excluded from the primary regression because the inferential question was conditional on intention: among students who already intended to exercise regularly, which post-intentional processes distinguished successful from unsuccessful enactment? Including non-intenders would have shifted the model from an action-control analysis of intention enactment to a broader prediction model of activity status in the full sample. Five nested models were estimated sequentially. Model 1 included sex and age. Model 2 added action planning and coping planning. Model 3 added habit automaticity. Model 4 added the interaction terms between habit automaticity and each planning construct. Model 5 added continuous intention strength. Continuous predictors were mean-centered before the interaction terms were created. Results are reported as odds ratios (ORs) with 95% confidence intervals (CIs). Nested models were compared using likelihood-ratio tests based on deviance differences, and model fit was summarized using log-likelihood, Akaike information criterion (AIC), Bayesian information criterion (BIC), and pseudo-R2 indices. Sensitivity analyses repeated the final adjusted logistic model under the three alternative intention-threshold specifications.

## 3. Results

### 3.1. Attrition Analysis, Psychometric Properties, and Zero-Order Associations

An attrition analysis compared students retained in the final analytic sample (*n* = 1670) with students who completed Wave 1 but were excluded from the final analysis because of incomplete data across waves (*n* = 112). As shown in Supplementary Table S6, retained and excluded participants did not differ significantly in age or Wave 1 habit automaticity. They differed significantly in sex distribution, although the effect size was small: excluded participants had a higher proportion of female students than the final analytic sample. These results suggest that attrition was limited and that the final analytic sample was broadly comparable to the initial Wave 1 sample on the available baseline characteristics, with the exception of a small sex-distribution difference.

Scale means indicated moderate-to-positive levels of intention and planning and moderate levels of habit automaticity (Table 1). Internal consistency was excellent for all focal constructs, with Cronbach’s alphas of 0.91 for habit automaticity, 0.93 for intention, 0.92 for action planning, and 0.90 for coping planning; composite reliability estimates ranged from 0.93 to 0.95. The CFAs strongly supported the distinction between action planning and coping planning. The two-factor planning model fit the data substantially better than the one-factor planning model, and the full four-factor model fit better than the three-factor model in which all planning items were combined (Supplementary Table S1). Standardized loadings were uniformly strong. These results justified treating action planning and coping planning as empirically distinct constructs in all subsequent analyses.

All focal psychological variables were positively associated with active status at Wave 3. Action planning showed the strongest bivariate association with Wave 3 activity status (r = 0.47), followed by intention (r = 0.47), habit automaticity (r = 0.40), and coping planning (r = 0.33; all *p* < 0.001). Intercorrelations among the psychological predictors were moderate to strong, especially between action planning and coping planning (r = 0.62) and between intention and action planning (r = 0.61). The correlation between intention and action planning was theoretically expected because action planning is a post-intentional self-regulatory process that is more likely to occur among students with stronger motivational commitment. However, this association did not indicate construct redundancy. The correlation of r = 0.61 corresponds to approximately 37% shared variance, leaving substantial nonshared variance between the constructs. In addition, the CFA results supported separating intention and action planning within the full four-factor model, and the measures were operationally distinct: intention assessed motivational commitment to perform regular leisure-time MVPA, whereas action planning assessed specific implementation details regarding when, where, what type, and how often the behavior would be performed. Taken together, the distinct item content, substantial nonshared variance, and four-factor CFA results provided evidence for the discriminant validity of intention and action planning in the present study.

### 3.2. Action-Control Profiles Under the Primary Intention Threshold

Using the primary threshold (mean intention ≥4.0), participants were classified into the four action-control groups shown in Table 2. In these results, active status refers to students who self-reported currently meeting the study’s regular leisure-time MVPA criterion; no separate continuous MVPA frequency, duration, or minutes-per-week variable was analyzed. Of the 1670 participants, 43.23% were successful intenders, 23.77% were unsuccessful intenders, 24.73% were non-intenders who were inactive, and 8.26% were non-intenders who were active despite not meeting the intention threshold. The intention-behavior gap among intenders was 35.48%. Additional group-level descriptives are summarized in Supplementary Table S2. Because the total numbers of female and male students differed, sex differences in the action-control profiles were examined descriptively using within-sex percentages rather than raw counts alone. The supplementary descriptives showed a pattern consistent with the adjusted models: female students were less likely than male students to be classified in the active groups overall, defined as successful intenders plus non-intenders who were active (42.69% vs. 58.71%). In brief, successful intenders showed the highest mean levels of action planning, habit automaticity, and intention, whereas non-intenders who were inactive showed the lowest levels on those same variables.

### 3.3. Hierarchical Logistic Regression Predicting Successful Enactment

Primary inferential analyses were conducted among participants classified as intenders under the primary threshold (*n* = 1119). Results of the five logistic regression models are shown in Table 3, with model-fit statistics and nested-model comparisons reported in Supplementary Table S3.

In Model 1, female students had lower odds of being active at Wave 3 than male students, whereas age was not associated with successful enactment. Adding action planning and coping planning in Model 2 significantly improved model fit over Model 1. Within this model, action planning was a strong positive predictor of successful enactment, whereas coping planning did not account for unique variance after adjustment for sex and age.

Model 3 further improved its fit by adding habit automaticity. In this model, habit automaticity emerged as a significant positive predictor, and action planning remained strongly associated with successful enactment. Coping planning again remained non-significant.

Model 4 tested whether habit automaticity moderated the effects of action planning and coping planning. Adding the two interaction terms did not improve model fit relative to Model 3, and neither interaction term was statistically significant. Consistent with these regression results, the predicted-probability plots in Supplementary Figures S1 and S2 show broadly parallel lines rather than visibly diverging functions.

Finally, Model 5 added continuous intention strength and again improved model fit. In the final model, stronger action planning, stronger habit automaticity, and stronger intention strength each independently predicted greater odds of being active at Wave 3, whereas coping planning did not show unique predictive value. Female students continued to have lower odds of successful enactment than male students, and age remained non-significant. The interaction terms also remained non-significant. The final model showed the strongest overall fit of the primary models (Nagelkerke R^2^ = 0.336).

Taken together, the primary-threshold analyses indicate that, among students who already intended to exercise regularly, successful enactment was prospectively distinguished by stronger action planning, stronger habit automaticity, and stronger intention strength. By contrast, coping planning did not add unique explanatory value once these variables were considered simultaneously.

### 3.4. Sensitivity Analyses

Sensitivity analyses examined whether the substantive conclusions depended on where the cutoff was placed for classifying participants as intenders. As shown in Supplementary Table S4, changing the threshold altered the descriptive size of the intention-behavior gap in the expected direction. The gap increased to 39.86% under the more inclusive threshold of mean intention >3.5, decreased to 17.66% under the stricter threshold of mean intention ≥5.0, and was 39.41% under the single-item threshold. Thus, the descriptive prevalence of successful and unsuccessful intenders depended partly on the cutoff used to define intenders, but this variation followed the expected pattern whereby stricter thresholds selected a more committed subset of intenders.

**Figure 2:**
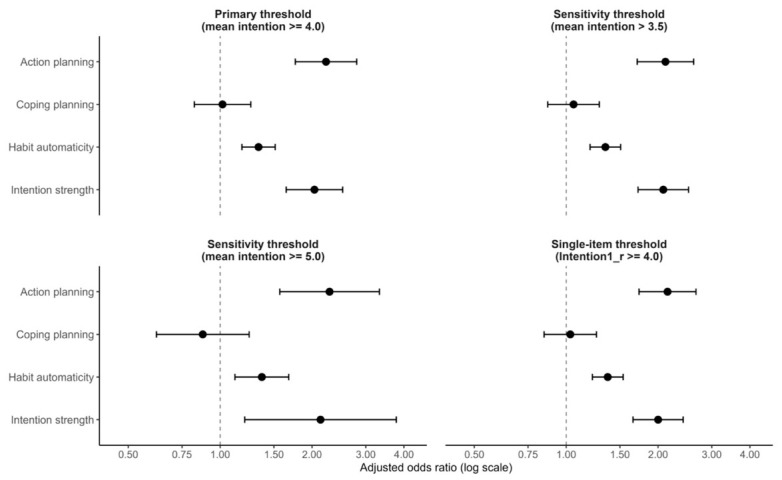
Forest Plot of Adjusted Odds Ratios Across the Primary and Sensitivity Models. Points represent adjusted odds ratios (ORs), and horizontal lines represent 95% confidence intervals. Results are drawn from the fully adjusted primary model and the fully adjusted sensitivity models. The primary model used the primary intention threshold of mean intention ≥4.0. Sensitivity models used mean intention >3.5, mean intention ≥5.0, and a single-item threshold of Intention 1 ≥ 4.0. Odds ratios greater than 1 indicate higher odds of being active (i.e., a successful intender) at Wave 3, whereas odds ratios less than 1 indicate lower odds. The figure displays only the focal psychological predictors to emphasize the robustness of the substantive pattern across thresholds. Sex and age were retained as covariates in all fitted models but are omitted from the figure for visual clarity.

Importantly, however, the regression pattern was highly stable across all three sensitivity specifications (Supplementary Table S5; Figure 2). Across the alternative thresholds, stronger action planning, stronger habit automaticity, and stronger intention strength each remained significant predictors of successful enactment, whereas coping planning remained non-significant. Female sex also remained a significant negative predictor, while age remained unrelated to the outcome. Neither planning x habit interaction was supported in any of the sensitivity models. Thus, although the descriptive size of the intention-behavior gap varied across threshold definitions, the substantive conclusion was robust: successful enactment was consistently characterized by stronger action planning, stronger habit automaticity, and stronger intention strength, whereas coping planning did not show unique prospective predictive value.

## 4. Discussion

### 4.1. Intention–Behavior Gap and Successful Self-Reported Exercise Enactment

This three-wave study examined which post-intentional processes distinguish successful from unsuccessful intenders in self-reported regular leisure-time MVPA enactment among Chinese undergraduates. Four findings were central. First, a substantial intention-behavior gap was observed under the primary threshold, with more than one-third of intenders failing to report meeting the regular-exercise criterion at follow-up. Second, among students who intended to exercise regularly, stronger action planning, stronger habit automaticity, and stronger intention strength prospectively distinguished those who later reported meeting the behavior criterion from those who did not. Third, coping planning did not show unique predictive value once action planning, habit automaticity, and intention strength were considered simultaneously. Fourth, the substantive pattern was robust across different cutoff scores used to classify participants as intenders. Taken together, these findings support an action-control account of student exercise behavior in which intention remains important but is insufficient without complementary regulatory and reflexive resources (Feil et al., 2023; Rhodes, 2021). Because the dependent variable was self-reported regular-exercise status, references to successful MVPA enactment in the Results and Discussion should be understood as meeting this stage-based regular-exercise criterion, rather than as objectively measured MVPA frequency, duration, intensity, or volume.

The active non-intender group also deserves brief theoretical consideration. Although this group was relatively small and was not central to the primary research question, it suggests that self-reported regular exercise can occur even when students do not meet the predefined intention threshold. Such cases may reflect established routines or habit-like automaticity, social or environmental facilitation, weak explicit intention combined with strong contextual cues, or measurement mismatch between the intention threshold and the stage-based behavior item. We did not analyze this group further because the primary inferential question concerned successful versus unsuccessful enactment among intenders; however, future studies could examine active non-intenders more directly to clarify when physical activity is maintained with relatively low conscious intention.

### 4.2. Relative Roles of Action Planning and Coping Planning

The most theoretically consequential finding was that action planning, rather than coping planning, emerged as the uniquely important planning variable among students who already intended to exercise regularly. This result fits the broader planning literature in one respect and departs from it in another. On the one hand, it is consistent with evidence that planning can help bridge the intention-behavior gap in physical activity and that both action planning and coping planning tend to show positive associations with activity behavior in aggregate (Carraro & Gaudreau, 2013). On the other hand, it contrasts with the recent meta-analytic structural equation modeling study by Wang et al., which suggested that coping planning may be the more important indirect pathway from intention to behavior (Wang et al., 2025). The present findings therefore suggest that the relative value of specific planning forms may be context-dependent.

In the present sample of university students, the immediate challenge may have been translating an already formed intention into a repeated, scheduled routine. Under this behavioral context, specifying when, where, what type of activity, and how often to exercise may have been more directly relevant than preparing for possible barriers in a general way. This interpretation does not imply that competing goals or environmental constraints are irrelevant to coping planning. Rather, in routine student exercise contexts, these constraints may first need to be managed through concrete scheduling and daily life organization, whereas coping planning may become more important when barriers are especially salient, disruptive, or personally difficult to overcome (Brown et al., 2024; Carraro & Gaudreau, 2013; Wang et al., 2025).

The null unique effect for coping planning should not be interpreted as evidence that coping planning is unimportant in general. Rather, it suggests that its role may be more contingent than that of action planning in this specific population, behavioral context, and analytic design. Coping planning may become especially important when barriers are salient, immediate, and personally consequential, such as during examination periods, schedule disruptions, facility constraints, poor weather, or competing academic demands. It may also be more predictive when students have sufficient self-efficacy or perceived behavioral control to implement barrier management strategies effectively. This possibility is consistent with Hou et al., who found that planning-related exercise processes depended on self-efficacy in Chinese students (Hou et al., 2022), and with Wang et al., whose meta-analytic model incorporated perceived behavioral control as an important upstream determinant (Wang et al., 2025). The present study did not directly assess barrier salience, self-efficacy, or perceived control; therefore, the findings should not be interpreted as a definitive test of when coping planning matters most.

In addition, the present linear logistic models cannot rule out the possibility that coping planning operates as a threshold, prerequisite, or nonlinear condition, such that some coping planning may be necessary for maintaining regular exercise, but higher levels beyond that point may not further increase enactment. In the present study, participants were already classified as intenders, and the target behavior was a clearly defined monthly regular-exercise pattern. Because the primary regression was restricted to intenders, the non-significant coping-planning finding should also not be generalized to non-intenders or to earlier transitions from non-intention to action, where barrier management may play a different role. Under those conditions, concrete scheduling may have been the more proximal requirement for success than generating obstacle-management strategies. Thus, the present findings qualify, rather than contradict, the emerging literature on coping planning by indicating that its unique role may vary across populations, designs, and behavioral contexts (Wang et al., 2025; Hou et al., 2022).

### 4.3. Habit Automaticity as a Reflexive Action-Control Resource

Habit automaticity also showed a robust independent association with successful enactment, even after planning and intention strength were entered into the model. This finding aligns closely with the M-PAC framework, which proposes that regulatory processes help preserve concordance between reflective motivation and behavior until reflexive processes such as habit begin to co-determine action control (Rhodes, 2021). It is also consistent with habit theory more broadly, which characterizes habit as context-linked automaticity that can sustain behavior with reduced deliberative effort once repetition has occurred in stable contexts (Gardner, 2015).

Conceptually, habit automaticity should not be equated with actual MVPA behavior; it captures the subjective automaticity with which leisure-time MVPA is initiated or performed, whereas regular-exercise status classifies whether students reported meeting a defined behavioral criterion. Importantly, the present findings do not imply that habit operates independently of reflective processes; rather, they indicate that habit automaticity contributed unique variance above and beyond planning and intention.

However, because baseline exercise behavior was not measured, this association should not be interpreted as evidence that habit automaticity predicted later regular-exercise status independently of prior exercise behavior. Habit automaticity is formed through repeated behavioral experience; therefore, Wave 1 habit automaticity may have partly reflected already established exercise routines or prior exercise history. In this sense, the results support an integrated perspective in which successful exercise enactment depends on both self-regulatory preparation and growing behavioral automaticity, a point that recent reviews have highlighted as increasingly important for the next generation of physical activity behavior change research (Rhodes, 2021; Gardner, 2015; Simpson et al., 2025).

A further notable finding was that continuous intention strength remained a significant predictor even within the subgroup classified as intenders. This pattern reinforces the view that classifying participants as intenders or non-intenders based on a cutoff score is useful for action-control classification, but it does not exhaust all meaningful variation in motivational commitment. Rhodes and Rebar argued that physical activity intention measures often combine both a decision component and a strength or commitment component (Rhodes & Rebar, 2017). The present results fit that argument well: once participants met the cutoff for being classified as intenders, those with stronger intentions were still more likely to enact them. This result also complements the broader intention-gap literature by showing that intention is neither sufficient nor irrelevant. Rather, intention continues to matter alongside post-intentional processes, which helps explain why the intention-behavior gap persists despite the predictive importance of intention itself (Feil et al., 2023; Rhodes & Rebar, 2017).

By contrast, the hypothesized habit x planning interactions were not supported. In the present data, habit automaticity and action planning appeared to function additively rather than multiplicatively. This result differs from Zhu et al., who reported a significant action planning x habit interaction in Chinese college students (Zhu et al., 2022), but it is not inconsistent with the broader literature, which has reported mixed evidence on whether habit weakens, strengthens, or leaves unchanged the role of deliberative predictors. Differences in study design may be relevant here. Zhu et al. used a two-wave moderated mediation approach focused on general exercise behavior, whereas the present study used a three-wave action-control design, a threshold-matched regular-exercise outcome, and complete-case logistic models among intenders only (Zhu et al., 2022). These design differences may have narrowed the question from general exercise correlates to the more specific problem of successful enactment among already motivated individuals. Under those conditions, main effects may be more stable than interactions. Accordingly, the present data favor the interpretation that action planning and habit automaticity each contribute to successful enactment without strong evidence that one changes the effectiveness of the other (Rhodes, 2021; Zhu et al., 2022).

### 4.4. Sex Differences in Successful Enactment

The sex effect in the present study also warrants comment as a distinct contextual finding. Female students were less likely than male students to be successful intenders across the primary and sensitivity models, whereas age was consistently unrelated to successful enactment. This pattern is consistent with broader evidence that insufficient physical activity is generally more prevalent among female than male adults globally and with recent evidence from Chinese university students showing lower high-volume MVPA participation among female students than male students (Strain et al., 2024; Deng et al., 2024). Thus, the observed sex difference provides some support for the external or nomological validity of the self-reported regular-exercise status outcome.

Importantly, female sex remained a significant negative predictor even after adjustment for action planning, coping planning, habit automaticity, and intention strength. This pattern suggests that the lower likelihood of successful enactment among female students may not be fully explained by the self-regulatory and reflexive processes measured in the present study. Instead, the intention–behavior gap for female students may also reflect external or contextual constraints that interfere with translating intention into regular exercise, such as unequal perceived access to exercise spaces, safety concerns, gendered social norms in campus sport or fitness settings, lower social support, or competing academic and daily life demands.

We caution against overinterpreting the mechanism of this effect because the present model did not include constructs that may help explain sex-linked disparities, such as perceived opportunity, social support, identity, safety, facility access, or gendered opportunity structures. However, this result is compatible with the broader student physical activity literature showing that enactment depends on contextual and social conditions, not only on motivation or planning. Future research should therefore test whether the lower odds of successful enactment among female students reflect differences in opportunity, competing demands, or other contextual constraints rather than weaker volitional processes per se (Brown et al., 2024).

### 4.5. Strengths, Limitations, and Future Directions

Several strengths increase confidence in the present conclusions. The study used a three-wave design, clearly separated the temporal ordering of habit automaticity, planning, and behavior, employed a behaviorally matched intention and outcome criterion, and tested robustness across multiple cutoff scores for classifying participants as intenders. The psychometric evidence was also strong: action planning and coping planning were empirically distinguishable, and all focal scales showed excellent reliability. These robustness analyses are especially important because intention measures in physical activity research often combine decisional and strength-related meaning; demonstrating stable conclusions across different intention cutoffs strengthens the defensibility of the main results (Rhodes & Rebar, 2017).

At the same time, several limitations should be acknowledged. First, all key variables were measured using self-report questionnaires, which may introduce recall bias, social desirability bias, and shared-method variance. This limitation is especially important for the dependent variable. Regular-exercise status was assessed using a single stage-based self-report item rather than objective physical activity indicators such as accelerometry, device-based MVPA minutes, or detailed activity logs. Therefore, the findings should be interpreted as explaining self-reported regular-exercise status rather than objectively measured MVPA frequency, duration, intensity, or total volume. The outcome was a stage-based active versus inactive indicator, which captures successful enactment or continuation of regular exercise but does not separate students who newly initiated regular exercise from those who were already maintaining regular exercise. Although this outcome was behaviorally matched to the intention items and was appropriate for the study’s action-control classification, it does not allow conclusions about objectively verified MVPA or compliance with guideline-based activity thresholds. This binary classification may also reduce behavioral information and limit comparability with studies using continuous MVPA frequency, duration, or volume outcomes. Another measurement consideration is that the focal predictors used different response scales; although these formats were retained to remain consistent with established measures, raw means and per-unit odds ratios should not be interpreted as directly comparable effect magnitudes across constructs. Future studies should incorporate device-based or diary-based physical activity measures to test whether the present action-control pattern generalizes objectively assessed MVPA. Future work should also test whether coping planning has nonlinear, threshold, or subgroup-specific effects, particularly among students facing salient barriers or lower perceived control.

A particularly important limitation is that baseline exercise behavior was not collected. Therefore, we could not conduct an additional analysis controlling for baseline exercise status, nor could we determine whether students were initiating regular exercise, maintaining an existing routine, or resuming a previous routine. This limitation is especially relevant for interpreting habit automaticity. Because habit automaticity develops through repeated performance in stable contexts, Wave 1 habit automaticity may have partly reflected already established exercise behavior rather than functioning as a behavior-free antecedent of later exercise status. Accordingly, the habit findings should be interpreted as prospective associations between baseline automaticity or established exercise routine strength and later self-reported regular-exercise status, rather than as evidence that habit automaticity predicts follow-up exercise independently of baseline behavior.

Intention and planning were measured in the same wave, so the study should not be interpreted as a formal mediation test. Finally, the use of complete-case analysis may have reduced precision if missingness was systematic, and the single-country undergraduate sample limits generalizability. These limitations suggest caution in causal interpretation, but they do not undermine the central action-control pattern observed here.

### 4.6. Practical Implications

The practical implications are reasonably clear. For university students who already want to exercise regularly, interventions may benefit more from helping them construct concrete action plans and repeat those plans in stable contexts than from assuming that additional motivational enhancement or general, non-specific obstacle-management planning alone will be sufficient. However, this implication should be interpreted in relation to the present study population and outcome. The findings do not suggest that coping planning should be abandoned, nor do they show that coping planning is unimportant for all students or under all conditions. Rather, among students who already intended to exercise regularly, concrete action planning may have been the more immediate self-regulatory requirement for reporting regular exercise at follow-up. A useful next step for intervention research would be to test whether action planning is especially effective early in the enactment process, whereas coping planning becomes more relevant under heightened barriers, lower perceived control, or transitions that disrupt routine.

Although the present sample was limited to Chinese undergraduates, the intention–behavior gap is not unique to this context; students in many countries face similar challenges in translating physical activity intentions into action, including time pressure, competing academic or work demands, social influences, screen-based routines, and variable access to supportive activity environments. What may differ across countries and education systems is the relative weight of these barriers. In the Chinese university context, academic expectations, campus living arrangements, peer norms, and structured educational schedules may shape when planning is feasible and whether repeated exercise can become habitual. Thus, the present findings should be viewed as both theoretically relevant to student physical activity more broadly and contextually conditioned by the cultural and educational environment in which the study was conducted.

More broadly, recent work in physical activity behavior change has emphasized the need to study behavior change more dynamically, to prioritize maintenance, and to better understand automatic processes. The present findings support those priorities by showing that, among students who already intended to exercise regularly, self-reported enactment was best characterized by strong intention, concrete action planning, and habit-like automaticity. Coping planning should still be understood as a volitional obstacle-management strategy, but it did not show unique predictive value in the present adjusted models (Brown et al., 2024; Simpson et al., 2025).

## 5. Conclusions

Among Chinese undergraduates who intended to exercise regularly, successful follow-through on a self-reported regular-exercise criterion was most consistently characterized by stronger action planning, stronger habit automaticity, and stronger intention strength. Coping planning did not show unique prospective predictive value, and no evidence was found that habit automaticity altered the usefulness of planning. The most defensible interpretation is therefore that self-reported regular exercise enactment in this context depends on a combination of commitment, concrete scheduling, and growing behavioral automaticity. These conclusions should not be interpreted as evidence regarding objectively measured MVPA volume, frequency, or intensity. Future research should extend this work by using objective or continuous MVPA indicators, including baseline exercise behavior, and testing whether coping planning has nonlinear or subgroup-specific effects, including among active non-intenders or students facing salient contextual barriers.

## Figures and Tables

**Table 1:** Descriptive Statistics, Reliability, and Zero-Order Correlations Among Key Variables.

No.	Variable	M	SD	α	CR	1	2	3	4	5
1	Habit automaticity	3.67	1.53	0.91	0.93	—				
2	Intention	4.26	1.16	0.93	0.95	0.55 ***	—			
3	Action planning	2.78	0.99	0.92	0.94	0.51 ***	0.61 ***	—		
4	Coping planning	2.51	0.97	0.90	0.93	0.48 ***	0.41 ***	0.62 ***	—	
5	Regular-exercise status (0/1)	0.51	0.50	—	—	0.40 ***	0.47 ***	0.47 ***	0.33 ***	—

Note. N = 1670. Habit automaticity was scored on a 1–7 scale, intention on a 1–6 scale, and action planning and coping planning on 1–5 scales, with higher scores indicating stronger levels of each construct. Because these constructs were measured on different response scales, their means should not be directly compared across constructs. Regular-exercise status was coded 0 = inactive and 1 = active; its mean therefore represents the proportion active. Correlations are Pearson coefficients; correlations involving regular-exercise status are point-biserial in interpretation. α = Cronbach’s alpha; CR = composite reliability. *** *p* < 0.001.

**Table 2:** Intention–Behavior Profiles Under the Primary Intention Threshold.

Action-Control Profile	*n*	% of Total Sample
Successful intender	722	43.23
Unsuccessful intender	397	23.77
Non-intender but active	138	8.26
Non-intender inactive	413	24.73

Note. N = 1670. Intenders were defined using the primary threshold of mean intention ≥4.0. Total intenders = 1119; total non-intenders = 551. The intention–behavior gap among intenders was 35.48%, calculated as unsuccessful intenders divided by all intenders.

**Table 3:** Hierarchical Logistic Regression Predicting Successful Enactment Among Primary-Threshold Intenders.

**Panel A. Adjusted Odds Ratios (95% Confidence Intervals)**					
Predictor	Model 1	Model 2	Model 3	Model 4	Model 5
Sex: Female vs. Male	0.46 *** [0.36, 0.60]	0.53 *** [0.40, 0.69]	0.60 *** [0.45, 0.80]	0.60 *** [0.45, 0.80]	0.65 ** [0.49, 0.87]
Age (years)	1.00 [0.88, 1.12]	0.97 [0.85, 1.11]	0.95 [0.83, 1.08]	0.94 [0.83, 1.08]	0.94 [0.82, 1.07]
Action planning (centered)	—	2.78 *** [2.27, 3.39]	2.61 *** [2.13, 3.20]	2.69 *** [2.17, 3.35]	2.22 *** [1.76, 2.80]
Coping planning (centered)	—	1.14 [0.95, 1.37]	1.01 [0.83, 1.22]	0.98 [0.80, 1.20]	1.02 [0.82, 1.26]
Habit automaticity (centered)	—	—	1.43 *** [1.27, 1.60]	1.43 *** [1.27, 1.60]	1.34 *** [1.18, 1.51]
Action planning × Habit automaticity	—	—	—	1.04 [0.90, 1.22]	1.03 [0.88, 1.22]
Coping planning × Habit automaticity	—	—	—	0.94 [0.81, 1.08]	0.95 [0.81, 1.10]
Intention strength (centered)	—	—	—	—	2.04 *** [1.65, 2.52]
**Panel B. Model Fit Statistics and Nested Comparisons**					
Statistic	Model 1	Model 2	Model 3	Model 4	Model 5
AIC	1424.84	1236.68	1199.68	1202.85	1159.76
BIC	1439.90	1261.78	1229.80	1243.02	1204.94
Nagelkerke R^2^	0.044	0.254	0.293	0.293	0.336
LR test vs. previous model	—	χ^2^(2) = 192.16, *p* < 0.001	χ^2^(1) = 39.00, *p* < 0.001	χ^2^(2) = 0.82, *p* = 0.662	χ^2^(1) = 45.09, *p* < 0.001

Note. Analyses were estimated among primary-threshold intenders (*n* = 1119). Entries in Panel A are odds ratios (ORs) with 95% confidence intervals. Males served as the reference category for sex. Continuous predictors were mean-centered before interaction terms were created; age was entered in years. Panel B reports model fit indices and likelihood-ratio tests comparing each model with the immediately preceding model. ** *p* < 0.01. *** *p* < 0.001.

## Data Availability

The data supporting the findings of this study are available from the corresponding author upon reasonable request. The data are not publicly available because they contain participant-level survey responses and are subject to ethical and privacy restrictions.

## Supplementary Materials

The following supporting information can be downloaded at: https://www.mdpi.com/article/10.3390/bs16060989/s1.
