# Supplementary material for: From Intention to Enactment: Action Planning and Habit Automaticity Distinguish Successful from Unsuccessful Intenders to Engage in Regular Leisure-Time Moderate-to-Vigorous Physical Activity
Source: Behav Sci (Basel). 2026 Jun 15;16(6):989. doi: 10.3390/bs16060989 (PMC13295560; doi:10.3390/bs16060989)
Supplement: Supplementary file 1 [file behavsci-16-00989-s001.zip › behavsci-4277909-supplementary.pdf]

Table S1. *Confirmatory Factor Analyses and Planning-Model Comparisons*

Panel A. Fit indices for planning and full measurement models

Model	χ^2	df	p	CFI	TLI	RMSEA	RMSEA 90% CI lower	RMSEA 90% CI upper	SRMR
Planning 1-factor	2163.56	14	$p < 0.001$	0.981	0.971	0.303	0.293	0.314	0.116
Planning 2-factor	117.23	13	$p < 0.001$	0.999	0.998	0.069	0.058	0.081	0.025
Full 3-factor (combined planning)	2828.02	74	$p < 0.001$	0.990	0.987	0.149	0.145	0.154	0.074
Full 4-factor (separate action and coping planning)	380.31	71	$p < 0.001$	0.999	0.999	0.051	0.046	0.056	0.028

Panel B. Key model-comparison deltas

Comparison	ΔCFI	ΔTLI	ΔRMSEA	ΔSRMR
Planning 2-factor minus Planning 1-factor	0.018	0.028	-0.234	-0.092
Full 4-factor minus Full 3-factor	0.009	0.011	-0.098	-0.046

Panel C. Standardized loadings for the preferred planning and full four-factor models

Model	Factor	Chinese item	English version	M (SD)	Std. loading
Planning 2-factor	Action planning	我有一个在每周什么时间段做中高强度运动的详细计划。	I have a detailed plan about when during the week I will perform MVPA.	2.70 (1.05)	0.861
Planning 2-factor	Action planning	我有一个每周在什么地方做中高强度运动的详细计划。	I have a detailed plan about where I will perform MVPA each week.	2.83 (1.14)	0.888
Planning 2-factor	Action planning	我有一个每周做什么中高强度运动的详细计划。	I have a detailed plan about what type of MVPA I will perform each week.	2.79 (1.14)	0.905
Planning 2-factor	Action planning	我有一个关于每周做几次中高强度运动的详细计划。	I have a detailed plan about how many times per week I will perform MVPA.	2.76 (1.10)	0.903
Planning 2-factor	Coping planning	我有一个关于，如果有事情干扰了我的运动计划后该如何去做的详细计划。	I have a detailed plan about what to do if something interferes with my exercise plan.	2.47 (1.07)	0.874
Planning 2-factor	Coping planning	我有一个如何应对可能影响我执行运动计划的事情的详细计划。	I have a detailed plan about how to deal with things that may affect my ability to carry out my exercise plan.	2.52 (1.07)	0.930
Planning 2-factor	Coping planning	我有一个关于在执行运动计划很困难的情况下该如何去做的详细计划。	I have a detailed plan about what to do when carrying out my exercise plan is difficult.	2.56 (1.08)	0.917
Full 4-factor (separate action and coping planning)	Habit automaticity	在闲暇时间做中高强度运动是：我自动就会去做的事情	Leisure-time MVPA is something I do automatically.	3.99 (1.74)	0.902
Full 4-factor (separate action and coping planning)	Habit automaticity	在闲暇时间做中高强度运动是：我不需要有意识的想着，就会去做的事情	Leisure-time MVPA is something I do without having to consciously remember.	3.62 (1.75)	0.918
Full 4-factor (separate action and coping planning)	Habit automaticity	在闲暇时间做中高强度运动是：我不需要想，就会去做的事情	Leisure-time MVPA is something I do without thinking.	3.64 (1.77)	0.857
Full 4-factor (separate action and coping planning)	Habit automaticity	在闲暇时间做中高强度运动是：常常在我还没意识到，就已经开始在做的事情	Leisure-time MVPA is something I start doing before I realize I am doing it.	3.44 (1.70)	0.806
Full 4-factor (separate action and coping planning)	Intention	我有意愿在未来一个月里，在我的闲暇时间做每周至少三次，每次至少 20 分钟的中高强度运动。	I intend to perform leisure-time MVPA at least three times per week, for at least 20 minutes each time, during the next month.	4.28 (1.29)	0.926

Full 4-factor (separate action and coping planning)	Intention	我计划在未来一个月里，在我的闲暇时间做每周至少三次，每次至少20 分钟的中高强度运动。	I plan to perform leisure-time MVPA at least three times per week, for at least 20 minutes each time, during the next month.	4.23 (1.27)	0.967
Full 4-factor (separate action and coping planning)	Intention	有多大的可能性，在未来一个月里，你能够在你的闲暇时间做每周至少三次，每次至少 20 分钟的中高强度运动？	How likely is it that, during the next month, you will be able to perform leisure-time MVPA at least three times per week, for at least 20 minutes each time?	4.30 (1.19)	0.895
Full 4-factor (separate action and coping planning)	Action planning	我有一个在每周什么时间段做中高强度运动的详细计划。	I have a detailed plan about when during the week I will perform MVPA.	2.70 (1.05)	0.867
Full 4-factor (separate action and coping planning)	Action planning	我有一个每周在什么地方做中高强度运动的详细计划。	I have a detailed plan about where I will perform MVPA each week.	2.83 (1.14)	0.880
Full 4-factor (separate action and coping planning)	Action planning	我有一个每周做什么中高强度运动的详细计划。	I have a detailed plan about what type of MVPA I will perform each week.	2.79 (1.14)	0.903
Full 4-factor (separate action and coping planning)	Action planning	我有一个关于每周做几次中高强度运动的详细计划。	I have a detailed plan about how many times per week I will perform MVPA.	2.76 (1.10)	0.909
Full 4-factor (separate action and coping planning)	Coping planning	我有一个关于，如果有事情干扰了我的运动计划后该如何去做的详细计划。	I have a detailed plan about what to do if something interferes with my exercise plan.	2.47 (1.07)	0.874
Full 4-factor (separate action and coping planning)	Coping planning	我有一个如何应对可能影响我执行运动计划的事情的详细计划。	I have a detailed plan about how to deal with things that may affect my ability to carry out my exercise plan.	2.52 (1.07)	0.929
Full 4-factor (separate action and coping planning)	Coping planning	我有一个关于在执行运动计划很困难的情况下该如何去做的详细计划。	I have a detailed plan about what to do when carrying out my exercise plan is difficult.	2.56 (1.08)	0.918

Note. All CFA models were estimated using WLSMV. Panel C shows standardized loadings for the preferred two-factor planning model and the preferred four-factor full model for brevity. Higher CFI and TLI and lower RMSEA and SRMR indicate better fit.

Table S2. *Additional Main-Threshold Group Profile Descriptives*

Panel A. Numeric descriptives by action-control group

Action-control group	n	Age, M (SD)	Habit automaticity, M (SD)	Intention, M (SD)	Action planning, M (SD)	Coping planning, M (SD)
Successful intender	722	20.09 (1.05)	4.47 (1.41)	5.10 (0.80)	3.38 (0.90)	2.91 (1.00)
Unsuccessful intender	397	20.07 (1.05)	3.50 (1.27)	4.46 (0.61)	2.59 (0.73)	2.37 (0.79)
Non-intender but active	138	19.96 (0.97)	3.19 (1.27)	3.15 (0.66)	2.48 (0.86)	2.37 (0.93)
Non-intender inactive	413	19.99 (0.93)	2.59 (1.21)	2.98 (0.73)	2.02 (0.70)	1.98 (0.77)

Panel B. Sex counts and within-group percentages

Action-control group	Sex	n (% within group)	% within sex
Successful intender	Female	249 (34.49)	33.11
Successful intender	Male	473 (65.51)	51.53
Unsuccessful intender	Female	211 (53.15)	28.06
Unsuccessful intender	Male	186 (46.85)	20.26
Non-intender but active	Female	72 (52.17)	9.57
Non-intender but active	Male	66 (47.83)	7.19
Non-intender inactive	Female	220 (53.27)	29.26
Non-intender inactive	Male	193 (46.73)	21.02

Table S3. *Main-Model Fit Statistics and Nested Likelihood-Ratio Tests*

Panel A. Fit statistics for the five main logistic models

Model	Parameters	Deviance	AIC	BIC	McFadden R^2	Cox & Snell R^2	Nagelkerke R^2
Model 1: covariates only	3	1418.84	1424.84	1439.90	0.025	0.032	0.044
Model 2: + action and coping planning	5	1226.68	1236.68	1261.78	0.157	0.185	0.254
Model 3: + habit automaticity	6	1187.68	1199.68	1229.80	0.184	0.213	0.293
Model 4: + planning x habit interactions	8	1186.85	1202.85	1243.02	0.185	0.213	0.293
Model 5: + continuous intention strength	9	1141.76	1159.76	1204.94	0.216	0.244	0.336

Panel B. Nested likelihood-ratio comparisons

Comparison	LR χ^2	df difference	p
Model 2 vs Model 1	192.16	2	< .001
Model 3 vs Model 2	39.00	1	< .001
Model 4 vs Model 3	0.82	2	= .662
Model 5 vs Model 4	45.09	1	< .001

Note. All models were estimated among primary-threshold intenders (n = 1,119). Fit statistics were computed directly from the generalized linear model components. Likelihood-ratio tests compare each model with the immediately preceding model in the hierarchical sequence

Table S4. *Sensitivity-Threshold Action-Control Profiles*

Threshold	Profile/summary	n	% of total sample
Mean intention > 3.5	Successful intender	774	46.35
Mean intention > 3.5	Unsuccessful intender	513	30.72
Mean intention > 3.5	Non-intender but active	86	5.15
Mean intention > 3.5	Non-intender inactive	297	17.78
Mean intention > 3.5	Total intenders	1287	—
Mean intention > 3.5	Total non-intenders	383	—
Mean intention > 3.5	Intention-behavior gap (%) among intenders	39.86	—
Mean intention ≥ 5.0	Successful intender	443	26.53
Mean intention ≥ 5.0	Unsuccessful intender	95	5.69
Mean intention ≥ 5.0	Non-intender but active	417	24.97
Mean intention ≥ 5.0	Non-intender inactive	715	42.81
Mean intention ≥ 5.0	Total intenders	538	—
Mean intention ≥ 5.0	Total non-intenders	1132	—
Mean intention ≥ 5.0	Intention-behavior gap (%) among intenders	17.66	—
Single-item threshold (Intention1_r ≥ 4.0)	Successful intender	758	45.39
Single-item threshold (Intention1_r ≥ 4.0)	Unsuccessful intender	493	29.52
Single-item threshold (Intention1_r ≥ 4.0)	Non-intender but active	102	6.11
Single-item threshold (Intention1_r ≥ 4.0)	Non-intender inactive	317	18.98
Single-item threshold (Intention1_r ≥ 4.0)	Total intenders	1251	—
Single-item threshold (Intention1_r ≥ 4.0)	Total non-intenders	419	—
Single-item threshold (Intention1_r ≥ 4.0)	Intention-behavior gap (%) among intenders	39.41	—

Note. "Intention-behavior gap (%) among intenders" was calculated as unsuccessful intenders divided by all intenders within each threshold definition.

Table S5. *Sensitivity-Threshold Final Logistic Models and Fit Statistics*

Panel A. Adjusted odds ratios (95% confidence intervals)

Predictor	Mean intention > 3.5	Mean intention ≥ 5.0	Single-item threshold
Sex: Female vs Male	0.71* [0.54, 0.92]	0.55* [0.34, 0.91]	0.65** [0.49, 0.85]
Age (years)	0.93 [0.82, 1.06]	0.97 [0.79, 1.20]	0.94 [0.83, 1.07]
Action planning (centered)	2.11*** [1.71, 2.62]	2.28*** [1.57, 3.32]	2.15*** [1.73, 2.66]
Coping planning (centered)	1.06 [0.87, 1.28]	0.88 [0.62, 1.24]	1.03 [0.85, 1.26]
Habit automaticity (centered)	1.34*** [1.20, 1.51]	1.37** [1.12, 1.68]	1.37*** [1.22, 1.54]
Action planning x Habit automaticity	1.03 [0.89, 1.20]	1.01 [0.80, 1.28]	1.01 [0.87, 1.18]
Coping planning x Habit automaticity	0.96 [0.83, 1.12]	0.89 [0.72, 1.10]	0.98 [0.84, 1.13]
Intention strength (centered)	2.08*** [1.72, 2.52]	2.13** [1.20, 3.78]	2.00*** [1.66, 2.42]

Panel B. Model fit statistics

Statistic	Mean intention > 3.5	Mean intention ≥ 5.0	Single-item threshold
n	1287	538	1251
AIC	1357.22	433.41	1299.63
BIC	1403.66	472.00	1345.81
Nagelkerke R^2	0.355	0.244	0.368

Note. All sensitivity analyses were estimated with threshold-specific intender subsamples. Panel A entries are odds ratios (ORs) with 95% confidence intervals from the final adjusted sensitivity models. Male served as the reference category for sex. Continuous predictors were mean-centered before interaction terms were created; age was entered in years. * $p < .05$. ** $p < .01$. *** $p < .001$.

Table S6. *Attrition Analysis Comparing Final Analytic Sample and Excluded Participants*

Characteristic	Final analytic sample, n = 1,670	Excluded participants, n = 112	Test statistic	p value	Effect size
Female sex, n (%)	752 (45.0%)	65 (58.0%)	$\chi^2(1) = 7.15$	.007	Cramér's V = .06
Age, M (SD)	20.05 (1.02)	20.02 (1.25)	t(1780) = 0.30	.768	d = 0.03
Wave 1 habit automaticity, M (SD)	3.67 (1.53)	3.61 (1.57)	t(1780) = 0.40	.688	d = 0.04

Note. Final analytic sample includes participants with complete data on all focal variables across the three survey waves. Excluded participants were those who completed Wave 1 but were not included in the final analytic sample because of incomplete data across waves. Values are n (%) unless otherwise indicated. Sex differences were examined using a chi-square test; age and Wave 1 habit automaticity were compared using independent-samples t tests. Effect sizes are reported as Cramér's V for sex and Cohen's d for continuous variables.

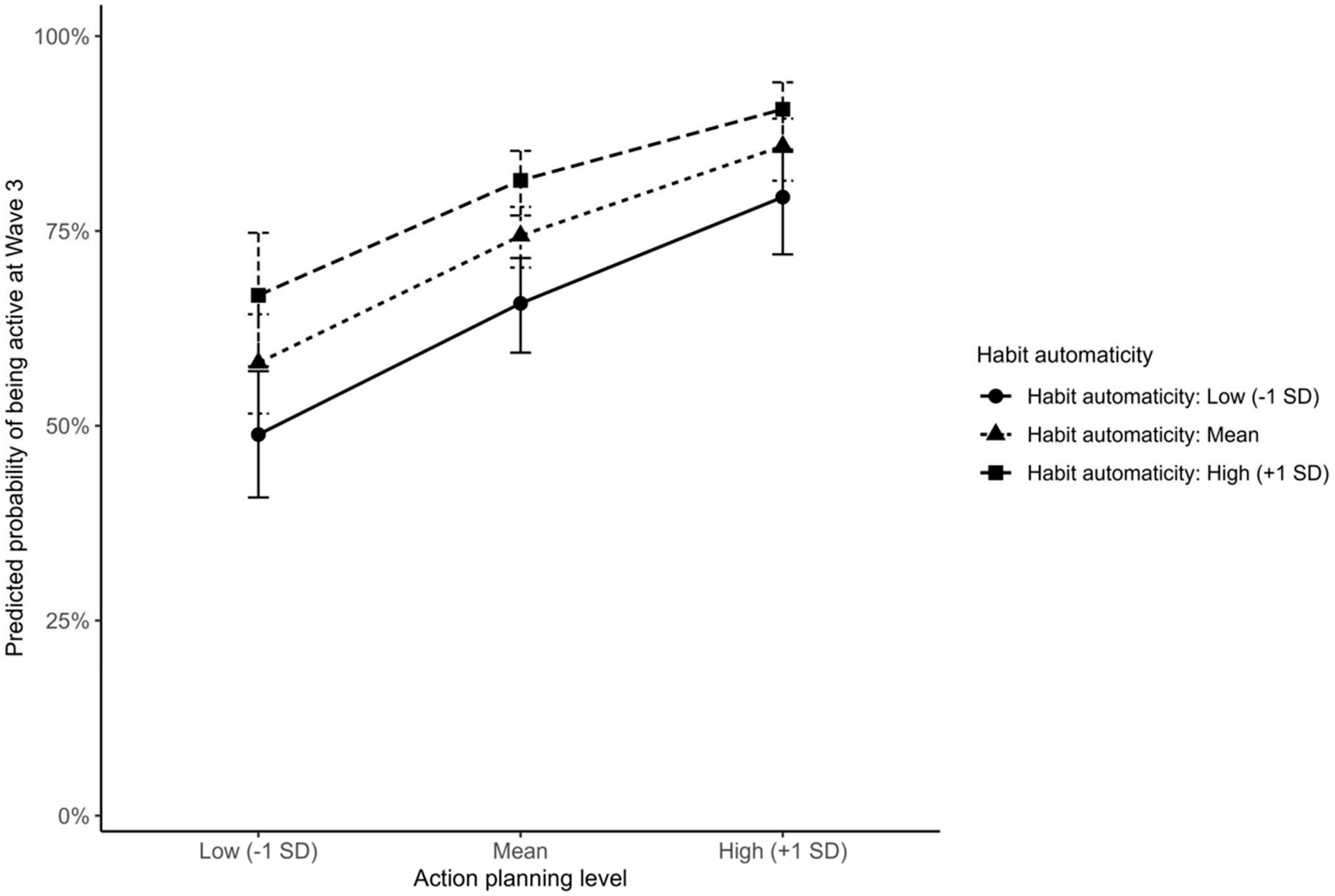

Figure S1. Predicted Probability Plot for the Action Planning × Habit Automaticity Interaction. Points represent model-based predicted probabilities of being active at Wave 3, and error bars represent 95% confidence intervals. Predicted values were generated from the fully adjusted primary-threshold interaction model. Habit automaticity levels correspond to low (−1 SD), mean, and high (+1 SD) values. Predictions were calculated with age held at the sample mean, sex set to the male reference category, intention strength held at its centered mean, and coping planning held at its centered mean. Because the Action Planning × Habit Automaticity interaction was not statistically significant, this figure is presented as a supplementary descriptive visualization of the fitted model.

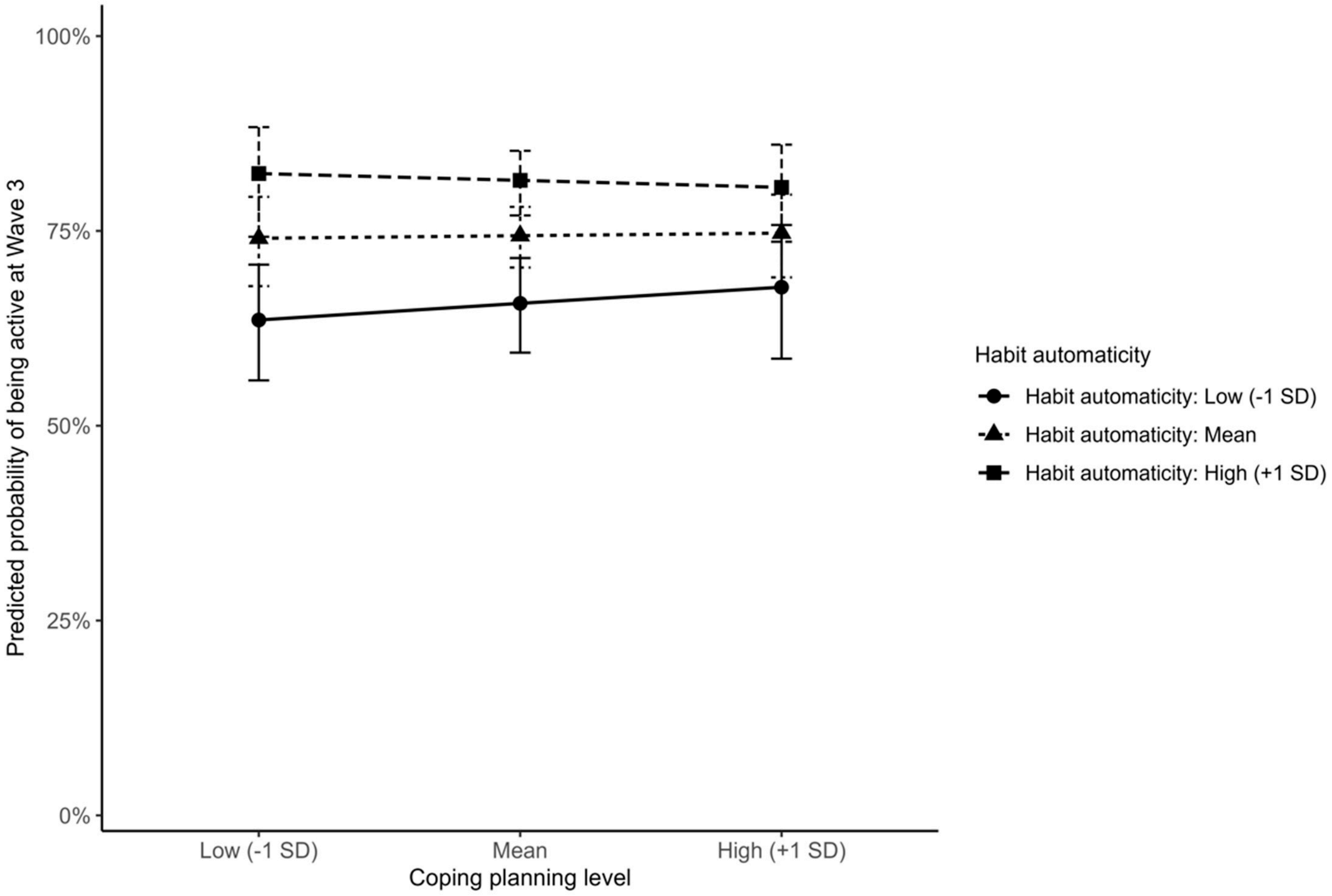

Figure S2. Predicted Probability Plot for the Coping Planning × Habit Automaticity Interaction. Points represent model-based predicted probabilities of being active at Wave 3, and error bars represent 95% confidence intervals. Predicted values were generated from the fully adjusted primary-threshold interaction model. Habit automaticity levels correspond to low (−1 SD), mean, and high (+1 SD) values. Predictions were calculated with age held at the sample mean, sex set to the male reference category, intention strength held at its centered mean, and action planning held at its centered mean. Because the Coping Planning × Habit Automaticity interaction was not statistically significant, this figure is presented as a supplementary descriptive visualization of the fitted model.
